# Cardiac magnetic resonance markers of pre-clinical hypertrophic and dilated cardiomyopathy in genetic variant carriers

**DOI:** 10.1186/s12916-025-04226-4

**Published:** 2025-07-15

**Authors:** Philip M. Croon, Marion van Vugt, Cornelis P. Allaart, Bram Ruijsink, Perry M. Elliott, Folkert W. Asselbergs, Rohan Khera, Connie R. Bezzina, Michiel Winter, A. Floriaan Schmidt

**Affiliations:** 1 Department of Cardiology, Amsterdam Cardiovascular Sciences, Amsterdam University Medical Centre, University of Amsterdam, Amsterdam, The Netherlands; 2 Section of Cardiovascular Medicine, Department of Internal Medicine, Yale School of Medicine, New Haven, CT USA; 3 Amsterdam Cardiovascular Sciences, Heart Failure and Arrhythmias, Amsterdam, The Netherlands; 4 Division Heart & Lungs, Department of Cardiology, University Medical Center Utrecht, Utrecht University, Utrecht, The Netherlands; 5 Institute of Cardiovascular Science, Faculty of Population Health, University College London, London, UK; 6 School of Biomedical Engineering & Imaging Sciences, King’s College London, London, UK; 7 Barts Heart Centre, St. Bartholomew’s Hospital, London, UK; 8 Centre for Heart Muscle Disease, Institute of Cardiovascular Science, University College London, London, UK; 9 Institute of Health Informatics, University College London, London, UK; 10 Biomedical Research Centre, National Institute for Health Research, University College London Hospitals, University College London, London, UK; 11 Section of Health Informatics, Department of Biostatistics, Yale School of Public Health, New Haven, CT USA; 12 Section of Biomedical Informatics and Data Science, Yale School of Medicine, New Haven, CT USA; 13 Center for Outcomes Research and Evaluation, Yale-New Haven Hospital, New Haven, CT USA; 14 Department of Experimental Cardiology, Amsterdam Cardiovascular Sciences, Amsterdam University Medical Centre, University of Amsterdam, Amsterdam, The Netherlands; 15 UCL British Heart Foundation Research Accelerator, London, UK

**Keywords:** Dilated cardiomyopathy, Cardiac magnetic resonance, Hypertrophic cardiomyopathy, Whole exome sequencing, Genetics

## Abstract

**Background:**

Patients with hypertrophic cardiomyopathy (HCM) and dilated cardiomyopathy (DCM) exhibit structural and functional cardiac abnormalities. We aimed to identify imaging biomarkers for pre-clinical cardiomyopathy in healthy participants carrying cardiomyopathy-associated variants (G +).

**Methods:**

We included 40,169 UK Biobank participants free of cardiac disease at the time of cardiac magnetic resonance imaging (CMR) and with whole exome sequencing. We validated 22 CMR measurements by associating them with incident atrial fibrillation (AF) or heart failure (HF). We subsequently assessed associations of these CMR measurements with HCM G+, DCM G + , or specific genes, utilising generalised linear models conditional on cardiac risk factors.

**Results:**

Thirteen CMR measurements were associated with incident AF and 15 with HF. These included left ventricular (LV) ejection fraction (EF; hazard ratio [HR] 0.61, 95% confidence interval [95%CI] 0.54; 0.69) for HF and indexed maximum left atrial volume (LAVi max; HR 1.47, 95%CI 1.29; 1.67) for AF. Five measurements associated with HCM G + , amongst which right ventricular (RV) end-systolic volume (RV-ESV; odds ratio [OR] 0.62, 95%CI 0.53; 0.74), RV-EF (OR 1.36, 95%CI 1.19; 1.55), and right atrial (RA) EF (OR 1.22, 95%CI 1.08; 1.39). Associations overlapping with incident disease and HCM G + had opposite effect directions, such as RV-ESV with HF (HR 1.22, 95%CI 1.07; 1.40). Two CMR measurements associated with DCM G + : LV-ESVi (OR 1.35, 95%CI 1.15; 1.58) and LV-EF (OR 0.75, 95%CI 0.64; 0.88). We observed significant associations with individual cardiomyopathy genes, finding that mitral annular plane systolic excursion associated with *TTN* and *TNNT2*, and LA pump volume and RA-EF associated with *MYH7*.

**Conclusions:**

We identified right-heart CMR measurements associated with HCM G + in healthy individuals, indicating early compensation of cardiac function. LV measurements associated with DCM G + , where CMR associations varied across individual DCM genes, suggesting distinct early pathophysiology.

**Supplementary Information:**

The online version contains supplementary material available at 10.1186/s12916-025-04226-4.

## Background

Cardiomyopathies are a group of disorders characterised by structural abnormalities of the heart which may lead to impaired cardiac function [1]. These abnormalities often contribute to the development of conditions such as heart failure (HF) and atrial fibrillation (AF) and can lead to sudden cardiac death [2, 3]. Hypertrophic cardiomyopathy (HCM) and dilated cardiomyopathy (DCM) are the two most common cardiomyopathies [4]. HCM is characterised by ventricular hypertrophy unexplained by abnormal loading conditions [2], whereas DCM is characterised by dilatation and impaired systolic function of the left ventricle or both ventricles [4].

Cardiomyopathies often have strong genetic aetiology, with pathogenic genetic variants found in up to 40% of HCM and DCM patients [1, 5, 6]. The prevalence of pathogenic variants in the general population depends on the variant adjudication, but has been estimated to be up to 1 in 150 for HCM and 1 in 250 people for DCM [7]. Disease onset is extremely variable across carriers of a cardiomyopathy (CMP) variant. In some, pathogenic variants lead to childhood disease, while others are affected late in life or not at all. The understanding of the early cardiac phenotype in individuals carrying a pathogenic genetic variant and are free of cardiac disease is currently limited [2, 5].

Cardiac magnetic resonance imaging (CMR) serves as the gold standard for non-invasive quantification of cardiac structure and function, and hence is the cornerstone in the routine monitoring of participants carrying CMP-associated variants (G +) and patients [1]. A relatively small number of predominantly left-heart CMR measurements, such as left ventricular (LV) ejection fraction (EF), end-diastolic volume (EDV), and LV wall thickness, are currently used in clinical care to monitor patients. Using genome-wide associations studies (GWAS), we and others have found that variants near CMP-causing genes, such as *TTN* and *BAG3*, associate with CMR measurements derived from healthy participants [8–10]. While the genetic associations suggest that CMR may contain valuable information for individuals carrying CMP-associated variants, GWAS exclude the rare causal CMP variants, and hence do not provide information on the association between CMR and G +.

In the current study, we leveraged data from 40,169 UK Biobank participants with available CMR data. We applied a DL-based automatic CMR analysis to derive 22 CMR measurements [11] and examined their association with participants carrying CMP-associated variants, as identified using whole exome sequencing (WES). First, we empirically validated the 22 derived CMR measurements by assessing their association with incident AF and HF. Next, we identified the subset of CMR measurements associating with 248 HCM G + and 144 DCM G +, as well as the CMR measurements associating with the most frequent genes for HCM and DCM. While previous studies have extensively explored the involvement of left-heart CMR measurements in CMP, we now present a more comprehensive evaluation also considering right-heart and atrial measurements. Furthermore, rather than focusing on family members, we uniquely explore associations in a general population setting amongst people without history of cardiac disease and cross-referenced these findings with previously identified common genetic CMP variants associating with CMR measurements. By identifying five CMR measurements associating with HCM G + and two with DCM G +, as well as identifying gene-specific associations, the current study provides an important evidence base for both machine-learning and more biologically orientated studies to explore pre-clinical CMP.

## Methods

### *Cardiomyopathy G* + 

G + were identified using the WES data available for 469,779 participants from the UK Biobank. Pathogenic and likely pathogenic variants for HCM and DCM were identified, as described by Bourfiss et al. [7]. Briefly, we selected genes classified to have definite, strong, or moderate evidence of pathogenicity as defined by Clinical Genome Resource (ClinGen) [12] and that were curated by Ingles et al. [13] and Jordan et al. [14]. Variants in the following genes were identified for HCM: *ACTC1*, *CSRP3*, *JPH2*, *MYBPC3*, *MYH7*, *MYL2*, *MYL3*, *TNNC1*, *TNNI3*, *TNNT2*, and *TPM1*, and for DCM we included *ACTC1*, *ACTN2*, *BAG3*, *DES*, *DSP*, *FLNC*, *JPH2*, *LMNA*, *MYH7*, *NEXN*, *PLN*, *RBM20*, *SCN5A*, *TNNC1*, *TNNI3*, *TNNT2*, *TPM1*, *TTN*, and *VCL* (Additional file 1: Table S1). Next, likely pathogenic and pathogenic variants in these genes were identified using the ClinVar NCBI-NIH database and the Dutch Society for Clinical Genetic Laboratory Diagnostics (Vereniging Klinische Genetische Laboratoriumdiagnostiek) database. Some variants were associated with both HCM and DCM. Excluding participants carrying these variants did not affect the associations with clinical outcomes [7] and were therefore included in both groups.

To account for conflicting submissions in ClinVar, we performed a sensitivity analysis by including only the genetic variants with consistent pathogenic classification across all submissions.

### UK Biobank participants

We utilised data from UK Biobank participants who enrolled to the CMR sub-study (*n* = 52,630). To account for a potential lag in registration or de novo diagnoses due to CMR, we excluded participants diagnosed with AF, HF, valvular disease, HCM, DCM, or CMP up to 30 days after the CMR (*n* = 2,888). Additional file 2: Methods detail the full data engineering strategy and Additional file 1: Table S2 lists the employed diagnosis codes. Ethics approval for the UKB study was obtained from the North West Centre for Research Ethics Committee (11/NW/0382) and all participants provided informed consent.

### CMR measurements derivation

**Fig. 1:**
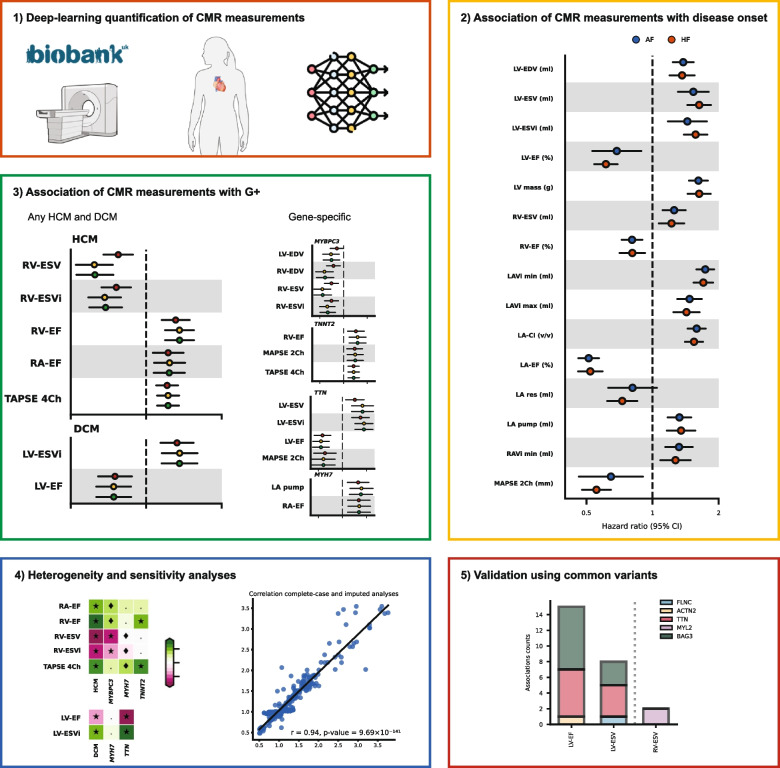
Study design to identify CMR measurements associated with HCM G + and DCM G +. Abbreviations: AF = atrial fibrillation, CMR = cardiac magnetic resonance imaging, DCM = dilated cardiomyopathy, G + = participants carrying disease-associated variants, HCM = hypertrophic cardiomyopathy, HF = heart failure

The full UK Biobank CMR protocol has been described in detail [15]. In short, all CMR examinations were performed on a 1.5 Tesla scanner (Magnetom Aera, Syngo Platform VD13A, Siemens Healthcare, Erlangen, Germany). We used a previously developed and validated DL model (AI-CMRQC) to extract 22 LV, right ventricular (RV), left (LA), or right atrial (RA) CMR measurements [11] (Fig. 1). CMRQC contains the following steps:

1. The quality of the CMR is evaluated and artefacts are rejected.
2. The full cycle of both short axis, 4- and 2-chamber is segmented by a 17-layer 2D fully convolutional network.
3. LV and RV volume curves and LV mass were calculated, from which cardiac function parameters including end-diastolic (EDV) and end-systolic volumes (ESV), stroke volume, and EF were derived.
4. In the output, quality control profiles of volume curves and LV/RV consistency were evaluated by support vector machine classifiers and inconsistencies were rejected or revised.

Cine images of short axis and 2- and 4-chamber long axis views were used to automatically calculate LV, RV, LA, and RA functional measurements, including biventricular EDV, ESV, stroke volume (SV), EF, LA/RA minimal and maximal volume (V min and V max), EF, 2- and 4-chamber mitral annular plane systolic excursion (MAPSE 2/4Ch), and 2- and 4-chamber tricuspid annular plane systolic excursion (TAPSE 2/4Ch). Several CMR measurements were indexed (i) for body surface area. To potentially inform clinical practice, we focused our analysis on CMR measurements that are either routinely available (such as EDV, ESV, SV, and EF), or can be easily extracted from standard clinical CMR images (such as atrial volumes, MAPSE, and TAPSE). We included both biventricular and atrial measurements to enable the discovery of novel biomarkers.

### Statistical analysis

To validate the CMR protocol, the association between CMR and the onset of AF and HF were evaluated using a Cox semi-parametric proportional hazards model. Participants without established disease at baseline were followed from their CMR appointment until the earliest of the following events: onset of AF or HF separate, lost to follow-up, death, or administrative censoring, with a maximum follow-up of 6.5 years. These models were adjusted for age (in years), male sex, hypertension, diabetes, smoking (ever/never), and hypercholesterolemia (Fig. 1). The model assumptions were evaluated by correlation of Schoenfeld residuals against time. Kaplan–Meier estimates of the cumulative AF or HF incidence were calculated by categorising CMR measurements into an 85% “reference” group and a 15% “risk increasing” group.

The marginal associations between CMR measurements and G + were ascertained using a generalised linear model with a binomial distribution and logit link function. To consider potential confounders, we created three models: model 1 was unadjusted, model 2 was adjusted for age and sex, and model 3 was additionally adjusted for cardiac risk factors: hypertension, diabetes, smoking status, and hypercholesterolaemia (Fig. 1).

We additionally explored potential differences in CMR association between men and women, and in age at CMR (< 60, 60–70, > 70). The interaction between subgroups was evaluated using likelihood ratio tests. Similarly, likelihood ratio tests were employed to explore possible non-linear associations between CMR measurements and HCM or DCM G + using restricted cubic splines (placing knots at the 15th, 50th, and 85th percentile).

The limited amount of missing data was imputed through Multiple Imputation by Chained Equations (MICE), creating ten datasets, and applying Rubin’s rules to combine estimates across imputed datasets. Results are presented as odds ratios (OR) or hazard ratios (HR), with 95% confidence intervals (95%CI). To account for multiple testing, we performed principal components analysis on the CMR measurements and identified the number of principal components explaining at least 90% of the variance. *p* values were evaluated against a multiplicity correction threshold of 0.05 divided by eight (6.25 × 10^−3^), representing the number of principal components necessary to explain at least 90% of the CMR variance (Additional file 2: Figs. S1 and S2). To assess the influence of multiple imputation on the associations of CMR measurements with incident AF, HF, and HCM G +, and DCM G +, we also performed a complete-case analysis and explored the Spearman correlation between the effect estimates of the imputed and complete-case analyses (Fig. 1).

The *Q*-test for heterogeneity was employed to determine to what extent the identified CMR associations with any G + differed across the individual HCM or DCM genes involved focusing on individual genes with at least 15 carriers (*MYBPC3*, *TNNT2*, and *MYH7* for HCM and *TTN* and *MYH7* for DCM). We furthermore set out to identify gene-specific CMR associations using the same approach as the main analysis considering any HCM or DCM variant per individual gene (Fig. 1).

### Genomics validation of CMR findings

Finally, for the subset of CMR measurements associating with G +, we sought to identify genetic support of this association by identifying common genetic variants located within or around known CMP variants and determining their association with these same CMR measurements (Fig. 1). Therefore, we leveraged CMR GWAS available in the NHGRI-EBI GWAS Catalog [16], extracted the genome-wide significant genetic variants, and determined which variants were located in a 1 megabase pair window around known HCM or DCM genes [8–10, 17–19].

## Results

CMR measurements and WES were available for 40,169 participants free of cardiac disease at the time of imaging. This included 248 (0.62%) participants with an HCM-associated variant and 144 (0.36%) with a DCM-associated variant. The most common genes associated with HCM were *MYBPC3* (45.6%), *TNNT2* (27.4%), or *MYH7* (19.4%), and with DCM *TTN* (30.6%), *MYH7* (23.6%), and *FLNC* (10.4%; Additional file 2: Fig. S3). The median age of the participants was 64 years (interquartile range [IQR] 58; 70), 47% was male, and the participants had a median LV-EF of 61% (IQR 57; 65) and RV-EF of 59% (IQR 55; 63; Table 1, Additional file 1: Table S3).

**Table 1:** Baseline characteristics of the UK Biobank participants with data on CMR measurements and whole exome sequencing

	*G − *	*HCM G* +	*DCM G* +
***Total sample size***	39,816	248	144
***Age (years)***	64.00 (58.00; 70.00)	63.00 (58.00; 69.00)	63.00 (58.00; 68.00)
***Sex***	18,714 (47.00)	122 (49.19)	69 (47.92)
***BMI (kg/m***^***2***^***)***	25.70 (23.33; 28.56)	25.51 (23.15; 28.02)	25.77 (23.33; 28.86)
***Hypertension***	7,277 (18.28)	45 (18.15)	27 (18.75)
***Diabetes***	417 (1.05)	5 (2.02)	2 (1.39)
***Smoking***	15,397 (38.67)	106 (42.74)	64 (44.44)
***Hypercholesterolaemia***	3,797 (9.54)	26 (10.48)	12 (8.33)
***Family history of heart disease***	16,661 (41.84)	117 (47.18)	63 (43.75)
***European ethnicity***	38,609 (96.97)	213 (85.89)	140 (97.22)
***LV-EDV (ml)***	140.66 (121.09; 164.11)	138.60 (116.94; 160.56)	147.36 (124.76; 163.70)
***LV-ESV (ml)***	54.26 (44.35; 66.89)	52.60 (42.14; 64.96)	56.04 (48.48; 69.75)
***LV-ESVi (ml)***	29.55 (24.96; 34.96)	28.81 (23.67; 34.16)	31.11 (26.05; 37.34)
***LV-SV (ml)***	85.61 (73.94; 99.10)	85.93 (72.54; 98.69)	85.83 (74.12; 97.24)
***LV-EF (%)***	61.04 (57.01; 65.14)	62.08 (58.06; 65.70)	59.03 (55.20; 63.47)
***LV mass (g)***	88.25 (73.79; 106.85)	86.54 (72.50; 105.35)	89.14 (70.83; 105.58)
***RV-EDV (ml)***	149.90 (127.84; 177.37)	143.99 (121.45; 173.23)	149.11 (127.74; 169.91)
***RV-ESV (ml)***	61.02 (49.42; 75.33)	57.15 (45.58; 68.63)	61.24 (48.80; 71.94)
***RV-ESVi (ml)***	33.20 (27.95; 39.23)	31.21 (26.16; 35.68)	33.55 (28.00; 37.61)
***RV-SV (ml)***	88.55 (75.95; 103.70)	86.94 (73.58; 104.14)	87.38 (73.62; 101.79)
***RV-EF (%)***	59.16 (55.30; 63.01)	60.29 (56.97; 64.63)	59.43 (55.18; 63.30)
***LAVi min (ml)***	13.08 (9.58; 16.99)	13.62 (9.62; 17.38)	13.31 (9.22; 17.29)
***LAVi max (ml)***	37.96 (31.66; 44.77)	39.57 (31.85; 46.22)	38.85 (32.23; 46.22)
***LA-CI (v/v)***	0.17 (0.13; 0.22)	0.18 (0.13; 0.22)	0.16 (0.12; 0.22)
***LA-EF (%)***	65.04 (59.79; 70.75)	65.69 (60.50; 70.41)	64.96 (59.99; 70.94)
***LA res (ml)***	20.91 (16.01; 26.22)	20.71 (16.71; 26.68)	20.44 (16.79; 25.55)
***LA pump (ml)***	22.41 (18.00; 27.42)	23.40 (17.56; 28.14)	24.72 (19.00; 28.21)
***RAVi min (ml)***	21.77 (17.12; 27.34)	20.66 (16.16; 26.79)	21.07 (17.35; 25.80)
***RAVi max (ml)***	44.63 (37.30; 53.49)	43.46 (35.93; 52.58)	44.53 (38.30; 53.62)
***RA-EF (%)***	50.32 (44.67; 56.55)	51.76 (46.16; 59.33)	51.52 (46.39; 58.66)
***MAPSE 2Ch (mm)***	16.45 (14.51; 18.56)	16.70 (14.46; 18.61)	15.95 (13.88; 18.37)
***TAPSE 4Ch (mm)***	12.42 (10.45; 15.13)	13.04 (11.21; 16.04)	12.62 (10.80; 15.16)

*Number (percentages) are given or median [IQR]. Abbreviations:* *CI* compliance index, *CMR* cardiac magnetic resonance imaging, *EDV* end-diastolic volume, *EF* ejection fraction, *ESV* end-systolic volume, *i* body surface area indexed, *LA* left atrial, *LV* left ventricular, *MAPSE 2Ch* mitral annular plane systolic excursion in 2-chamber view, *pump* pump volume, *RA* right atrial, *res* reservoir volume, *RV* right ventricular, *SV* stroke volume, *TAPSE 4Ch* tricuspid annular plane systolic excursion in 4-chamber view, *Vi max* maximum indexed volume, *Vi min* minimum indexed volume

### CMR associations with the onset of AF and HF

**Fig. 2:**
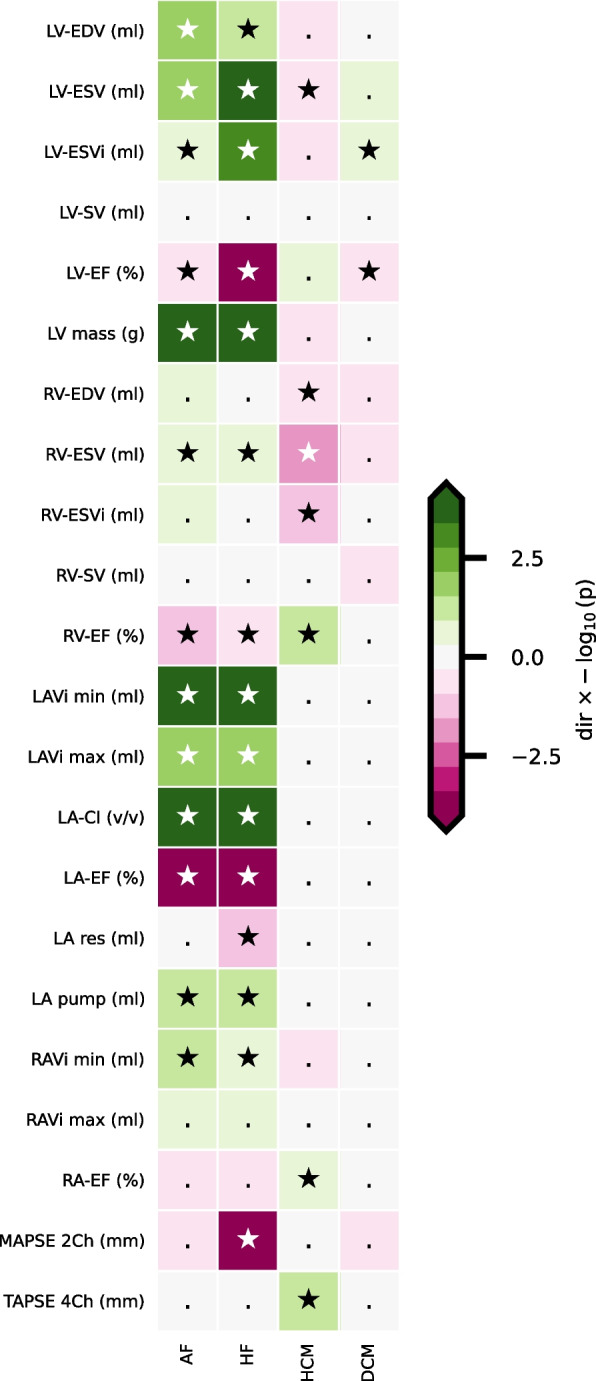
Association of CMR measurements with incident atrial fibrillation or heart failure and carriership of genetic variants associated with hypertrophic cardiomyopathy or dilated cardiomyopathy. Associations are presented as − log_10_(*p* value) multiplied by the effect direction. Significant results, as defined by the Bonferroni-corrected *p* value threshold of 6.25 × 10^−3^, are indicated with a star. Abbreviations: AF = atrial fibrillation, CI = compliance index, CMR = cardiac magnetic resonance imaging, DCM = dilated cardiomyopathy, EDV = end-diastolic volume, EF = ejection fraction, ESV = end-systolic volume, HCM = hypertrophic cardiomyopathy, HF = heart failure, i = body surface area indexed, LA = left atrial, LV = left ventricular, MAPSE 2Ch = mitral annular plane systolic excursion in 2-chamber view, pump = pump volume, RA = right atrial, res = reservoir volume, RV = right ventricular, SV = stroke volume, TAPSE 4Ch = tricuspid annular plane systolic excursion in 4-chamber view, Vi max = maximum indexed volume, Vi min = minimum indexed volume

**Fig. 3:**
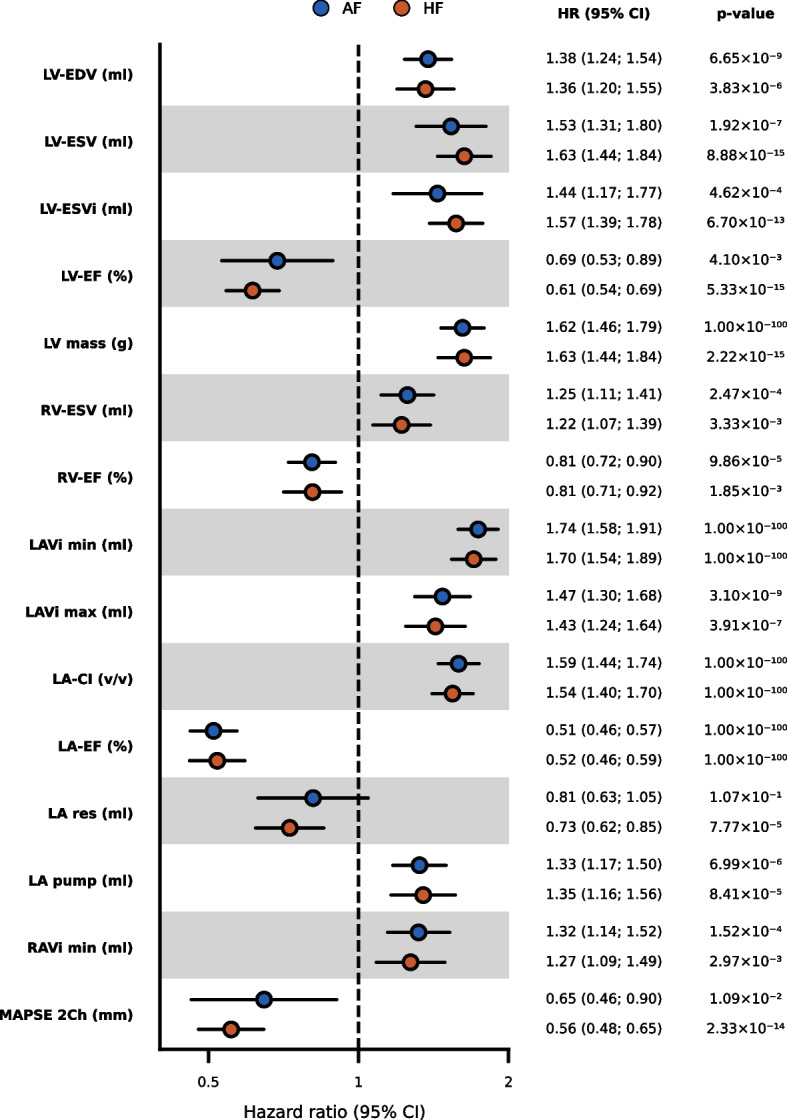
Hazard ratios for the association of CMR measurements with incident atrial fibrillation or heart failure. Effect magnitudes are presented per standard deviation increase in CMR measurements. Abbreviations: AF = atrial fibrillation, CI = compliance index, CMR = cardiac magnetic resonance imaging, EDV = end-diastolic volume, EF = ejection fraction, ESV = end-systolic volume, HF = heart failure, HR = hazard ratio, i = body surface area indexed, LA = left atrial, LV = left ventricular, MAPSE 2Ch = mitral annular plane systolic excursion in 2-chamber view, RA = right atrial, RV = right ventricular, SV = stroke volume, TAPSE 4Ch = tricuspid annular plane systolic excursion in 4-chamber view, Vi max = maximum indexed volume, 95% CI = 95% confidence interval

The 22 DL-derived CMR measurements were first empirically validated by determining their association with incident AF (*n* = 646) and HF (*n* = 202). The median follow-up was 2.06 years (IQR 1.18; 3.31) for AF cases and 3.33 years (IQR 2.47; 4.80) for their controls, and 2.37 years (IQR 1.37; 3.86) for HF cases and 3.33 years (IQR 2.47; 4.81) for their controls and 87 participants were diagnosed with both AF and HF. Adjusted Cox models identified 13 measurements associated with AF and 15 with HF (Figs. 2 and 3, Additional file 1: Tables S4 and S5). For example, per standard deviation increase of LV-EF, the HR was 0.69 (95%CI 0.53; 0.89) for incident AF and 0.61 (95%CI 0.54; 0.69) for incident HF. For RV-EF, this was 0.81 (95%CI 0.72; 0.90) for AF and 0.81 (95%CI 0.71; 0.92) for HF, and for LA-EF this was 0.51 (95%CI 0.46; 0.57) for AF and 0.52 (95%CI 0.46; 0.59) for HF. Furthermore, we observed that larger values of LV-EDV, LV-ESV, LV-ESVi, LV mass, RV-ESV, LA-Vi, LA compliance index, LA pump volume, and RA-Vi increased the risk of developing both AF and HF. An increase in LA reservoir volume (OR 0.73, 95%CI 0.62; 0.85) and MAPSE 2Ch (OR 0.56, 95%CI 0.48; 0.65) was associated with a decreased risk of HF but not AF (Figs. 2 and 3, Additional file 1: Tables S4 and S5). The Kaplan–Meier analysis is depicted in Additional file 2: Figs. S4 and S5.

### *CMR associations with G* + 

**Fig. 4:**
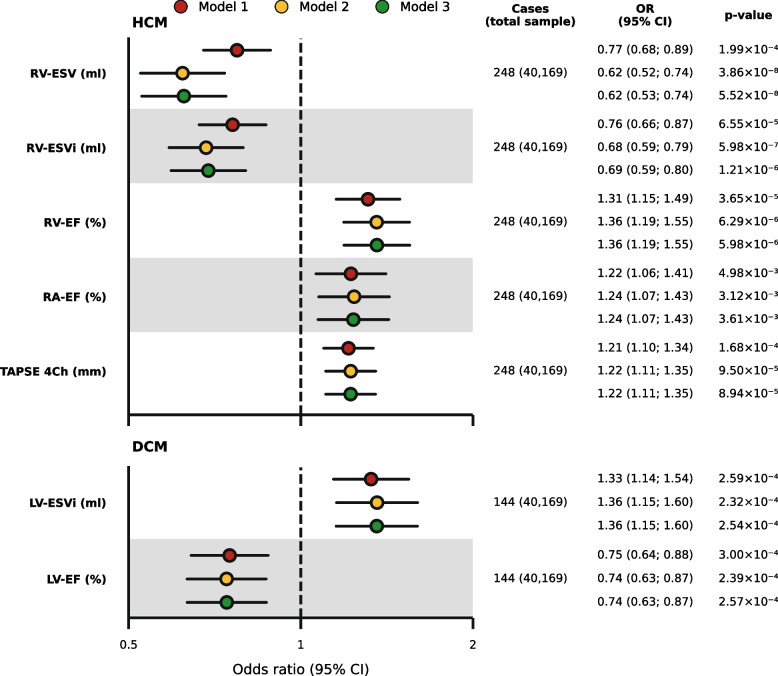
Odds ratios for the association of CMR measurements with hypertrophic cardiomyopathy or dilated cardiomyopathy G +. Model 1 is univariable, model 2 is adjusted for age and sex, and model 3 is adjusted for age, sex, and the comorbidities hypertension, diabetes, hypercholesterolaemia, and smoking. Abbreviations: CMR = cardiac magnetic resonance imaging, DCM = dilated cardiomyopathy, EF = ejection fraction, ESV = end-systolic volume, G + = participants carrying disease-associated variants, HCM = hypertrophic cardiomyopathy, i = body surface area indexed, LV = left ventricular, RA = right atrial, RV = right ventricular, OR = odds ratio, TAPSE 4Ch = tricuspid annular plane systolic excursion in 4-chamber view, 95% CI = confidence interval

We identified five CMR measurements associated with HCM G + and two CMR measurements associated with DCM G + after adjusting for age, sex, and known cardiac risk factors (Fig. 4). We focus on the fully adjusted model, because the results were consistent across models with fewer or no covariates. The odds of HCM G + increased with larger values of RV-EF (OR 1.36, 95%CI 1.19; 1.55), RA-EF (OR 1.24 95%CI 1.07; 1.43), and TAPSE 4Ch (OR 1.22, 95%CI 1.11; 1.35) and decreased with RV-ESV (OR 0.62, 95%CI 0.53; 0.74) and RV-ESVi (OR 0.69, 95%CI 0.59; 0.80; Fig. 4, Additional file 1: Table S6). The odds of DCM G + decreased with larger values of LV-EF (OR 0.74, 95%CI 0.63; 0.87) and increased with LV-ESVi (OR 1.36, 95%CI 1.15; 1.60; Fig. 4, Additional file 1: Table S7). Comparing these results to the CMR associations with incident AF and HF, we found that associations with DCM G + were typically in the same direction as that of HF and AF associations. However, for HCM G +, we generally observed that CMR measurements with incident disease were in the opposite direction. For example, larger values of RV-EF increased the odds of HCM G + but were associated with a decreased risk of AF and HF (Fig. 2).

**Fig. 5:**
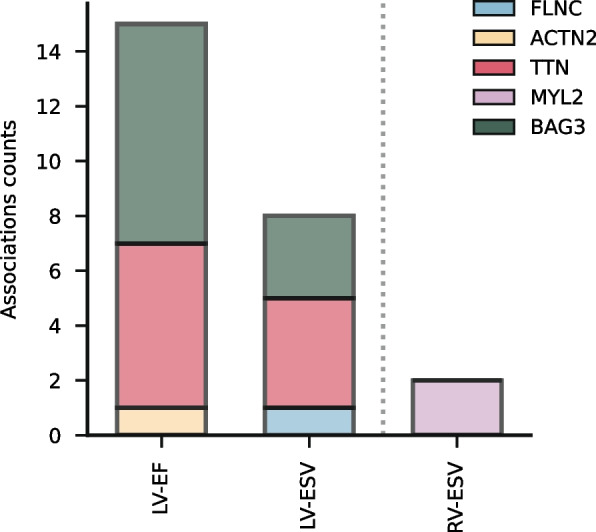
Frequency of genetic variants in cardiomyopathy-associated genes associating with CMR measurements. Genetic variants were selected within 1 megabase pair of cardiomyopathy-associated genes and searched in genome-wide association study summary statistics. Associations with dilated cardiomyopathy are depicted left of the vertical line, those with hypertrophic cardiomyopathy to the right. Abbreviations: EF = ejection fraction, ESV = end-systolic volume, LV = left ventricular, RV = right ventricular

The Spearman correlation between the complete-case analysis results and results based on multiple imputed data was 0.94 (*p* value 9.69 × 10^−141^; Additional file 2: Fig. S6, Additional file 1: Tables S4–S8). Sensitivity analyses did not identify substantial evidence for non-linearity, or effect modification by age or sex (Additional file 1: Tables S9–S11). Restricting the included variants to only pathogenic or likely pathogenic submissions did not alter any of the associations (Additional file 1: Tables S6 and S7).

### Gene-specific CMR associations

We then focused on pathogenic and likely pathogenic variants in the HCM and DCM genes with at least 15 carriers (*MYBPC3*, *TNNT2*, and *MYH7* for HCM and *TTN* and *MYH7* for DCM). Subsequently, we assessed whether CMR measurements specifically associated with variants in these six individual genes. In addition to the overall associations with HCM, we found that *TNNT2* (*n* = 68) uniquely associated with MAPSE 2Ch (OR 1.41, 95%CI 1.11; 1.79), and *MYBPC3* (*n* = 113) associated with LV-EDV (OR 0.70, 95%CI 0.55; 0.90) and RV-EDV (OR 0.58, 95%CI 0.45; 0.75). The associations of LV-EF and LV-ESVi with DCM G + showed substantial heterogeneity, which were driven by variants in *TTN* (Additional file 2: Figs. S7 and S8, Additional file 1: Tables S12 and S13). For DCM, we identified specific CMR associations with *TTN* (*n* = 44; MAPSE 2Ch OR 0.58, 95%CI 0.41; 0.81) and *MYH7* (*n* = 34; LA pump volume OR 1.70, 95%CI 1.21; 2.38 and RA-EF OR 1.61, 95%CI 1.16; 2.23; Additional file 2: Figs. S9 and S10, Additional file 1: Table S13).

### Genomic validation of identified CMR biomarkers

To further validate our findings, we identified common genetic variants located within and around known HCM and DCM genes that were associated with the subset of CMR measurements linked to G +. For this, we extracted genome-wide significant findings from GWAS conducted on biventricular EF and ESV, RV-EDV, RV-SV, and RA-EF. We found that 15 genetic associations of variants near or within causal genes for DCM (*BAG3*, *TTN*, and *ACTN2*) also associated with LV-EF (Fig. 5, Additional file 1: Table S14). Similarly, eight genetic associations near known DCM genes were observed for LV-ESV (Fig. 5). For CMR measurements associating with HCM G +, we were able to genetically validate the association of RV-ESV, finding two genetic associations within/near *MYL2* associating with this measurement. A similar validation step could not be conducted for TAPSE because this measurement has not yet been considered in GWAS.

## Discussion

In the current study, we empirically validated DL-based automatic analysis of 22 CMR measurements by confirming associations with incident AF and HF. We subsequently combined these measurements with WES data to establish imaging biomarkers of the left and right ventricle and the right atrium in participants carrying variants associated with HCM or DCM. Focusing on the most common genes associated with HCM and DCM, we identified CMR associations with genetic variants in specific genes. Lastly, we provide genetic validation, confirming that common genetic variants within or around CMP-causing genes were associated with the same CMR measurements. These validated CMR measurements offer insights into the pre-clinical phenotypes of CMP and provide potential surrogate endpoints for clinical trials evaluating novel therapeutics [20, 21]. Compared to other studies on early imaging markers for CMP G + [22–24], our study wholistically considers both left- and right-heart measurements. Additionally, rather than focusing on high-risk family members of CMP patients, our study uniquely explores CMR associations in participants from the general population, who are not subjected to the same level of cardiac screening offered to CMP patient family members. We furthermore identify relevant gene-specific associations between CMR measurements and CMP G +, suggesting that pre-clinical cardiac function may differ depending on the affected gene. In addition, our study assesses the association of CMR measurements with incident AF and HF, enabling assessment of early cardiac phenotype changes in a way that is directly relevant for long-term cardiovascular risk.

Of the 22 CMR measurements, 13 associated with incident AF and 15 with HF, confirming subtle structural and functional cardiac abnormalities in these diseases. Known CMR measurements associating with AF and HF included LV-EF, LV mass, RV-ESV, RV-EF, and LA volume. All measurements associated with AF also associated with HF in the same direction. These results strongly support that DL-based automatic analysis of CMR is a feasible modality for risk stratification in early cardiac disease.

Next, we established novel CMR measurements that associate with carriership of variants associated with HCM and DCM G +. Primarily right-heart measurements (RV-EF, RV-ESV, RV-ESVi, RA-EF, and TAPSE 4Ch) associated with HCM G +. While HCM in patients is typically characterised by LV hypertrophy [2], our findings suggest that RV hypercontractility might be a feature of HCM G + without cardiac disease. The absence of an association with LV hypertrophy in our study may reflect a lack of statistical power or indicate that functional changes precede structural remodelling. The role of right-heart characteristics in early or pre-clinical HCM remains less well-characterised, though RV measurements have previously been found to be key predictors of HCM progression, associating with an increased risk of supraventricular and ventricular arrhythmias, progressive HF, and sudden cardiac death [20, 21]. This aligns with pathogenic sarcomeric variants leading to hypercontractility and increased myofilament sensitivity to calcium [25, 26]. These variants likely exert their effects on both ventricles, where the thin-walled RV may be particularly susceptible to early contractile abnormalities [27]. Thus, the observed right-heart associations in HCM G + might indicate that RV hypercontractility serves a similar etiological role in HCM as LV hypercontractility, which was shown to be related to HCM [18]. While the imaging biomarkers commonly utilised in the management of HCM are primarily those related to LV mass, we demonstrated that RV measurements are affected in HCM G + and may play a role in monitoring early disease. RV measurements are measured routinely in clinical practice, but despite research suggesting that RV involvement impacts disease presentation and prognosis, they are not yet incorporated into clinical guidelines for HCM management [1, 28–30]. Moreover, prior research found that the decline of right ventricular function precedes the left ventricle in HCM patients [21]. CMR measurements not only enhance disease diagnosis and risk stratification, but may also serve as surrogate markers for therapeutic efficacy, such as with mavacamten [31]. By identifying additional CMR measurements associated with CMP G +, we have taken an initial step toward establishing possible surrogate endpoints for future clinical trials.

The associations with RV-EF and RV-ESV were in opposite effect directions compared to those in AF and HF: where a standard deviation increase in RV-EF decreased risk of incident AF and HF, it increased the odds of HCM G +. Similar directional discordance was observed in GWAS of HCM and DCM, where genetic correlation demonstrated that LV-ESV correlated negatively with HCM (− 0.31) but positively with DCM (0.46) [18]. We hypothesised that RV hypercontractility could be an early feature of HCM G +, due to pathogenic variants affecting the thin-walled RV. In contrast, RV dysfunction in AF and HF is more likely to reflect maladaptive remodelling and advanced disease secondary to chronically increased left-sided filling pressures and pulmonary hypertension [32–34]. These results broaden the effect of proposed therapeutic strategies targeting contractility in sarcomeric variants [35]. Furthermore, only a subset of the CMR measurements associated with CMP G + were also associated with AF and HF onset. As such, carriership of these variants may not simply reflect early signs of AF or HF, but instead represents a unique pre-clinical phenotype otherwise overlooked.

LV-EF and LV-ESVi were associated with DCM G + in the same direction as the associations with AF and HF. LV-EF is a strong predictive marker for HF in DCM and together with LV-ESVi the most frequently used imaging marker for DCM diagnosis and monitoring [36]. We observed considerable heterogeneity in the most common genes associated with DCM and showed that *TTN* drives the observed association with LV-EF and LV-ESVi. *TTN* encodes a giant elastic protein and pathogenic *TTN* variants are known to impair myocardial contraction [37], which is reflected in the observed associations with reduced LV-EF and MAPSE in the gene-specific analysis. Pathogenic *MYH7* variants associated with DCM impair sarcomeric function due to reduced actin-activated ATPase, leading to energetically inefficient contraction [38]. Whereas no prior associations with atrial function have been reported, our study reveals novel associations of *MYH7* with increased LA pump volume and RA-EF. In HCM, we observed gene-specific effects for *MYBPC3*, associated with reduced LV-EDV, RV-EDV, and RV-ESVi, and *TNNT2*. Pathogenic variants in *MYBPC3* contribute to hypercontractility and impaired relaxation [25, 26, 39], consistent with the reduced ventricular volumes we observed. *TNNT2* regulates myofilament calcium sensitivity and hypercontractility [39] and its association with increased RV-EF, MAPSE, and TAPSE in our study indicates increased biventricular systolic performance. The limited overlap in CMR associations between *TNNT2* and *MYBPC3* may support previous observations that HCM patients with thin filament pathogenic variants have different phenotypic expression compared to those with thick filament variants [39]. These results indicate that the pre-clinical manifestations of variants in specific genes are heterogeneous, and it may be beneficial to consider these differences in monitoring and treating CMP G + and patients. This work provides an initial step in identifying these differences and provides further support for more systematic exploration of gene-specific association required for translation to clinical practice.

Potential limitations should be acknowledged. This study focused on identifying CMR measurements associating with G + and exploring associations with AF and HF incidence. We were unable to confirm whether the identified CMR measurements also associate with cardiac disease onset in G +, because of the moderate CMP G + sample size and limited follow-up after CMR measurements. While the UK Biobank has substantial follow-up after first enrolment, the imaging sub-study is still currently inviting participants, and as such the follow-up since CMR measurement is considerably shorter (median 3.34 years, IQR 2.48; 4.82) prohibiting a meaningful analysis of disease onset of the rare cardiomyopathies. Confirmatory studies and further prospective validation of our findings are warranted, which may be especially relevant for HCM G +, because the association with right-heart measurements is less well established. Associations between CMR and AF or HF were considered to (1) validate the accuracy of the derived CMR measurements and (2) provide the necessary context to interpret the CMP G + associations. In addition to the identified imaging biomarkers for pre-clinical HCM and DCM phenotypes, larger sample size research is needed to explore the relevance of non-CMR measurements such as general patient characteristics or electrocardiography measurements. Integrating factors from multiple modalities can help derive future classification models to identify disease-free individuals who are likely to be CMP G +. Additionally, our dataset predominantly comprises individuals of European ethnicity, potentially limiting the generalisability of our findings to more diverse populations.

## Conclusions

In conclusion, right-heart measurements are associated with HCM G +, while LV measurements associate with DCM G + in individuals without established cardiac disease. The identified RV and RA measurements associating with HCM G + reflect enhanced cardiac function, potentially indicating transient compensatory mechanisms. The observed variability in CMR associations with DCM genes suggests that early cardiac phenotype differs by individual genes, which is in line with current understanding of clinical manifestations being gene specific.

## Supplementary Information

Additional file 1: Tables S1–S14. Table S1 Genes with pathogenic and likely pathogenic variants for hypertrophic cardiomyopathy or dilated cardiomyopathy. Table S2 Disease definitions based on ICD-10 codes and self-reported codes. Table S3 Baseline table for participants of the UK Biobank without established cardiac diseaseat the time of cardiac imaging visit. Table S4 Hazard ratio estimates for the association between CMR measurementsand the onset of atrial fibrillation and heart failure in people without established cardiac disease at the time of their imaging visit. Table S5 Hazard ratio estimates for the association between binary CMR measurementsand the onset of atrial fibrillation and heart failure in people without established cardiac disease at the time of their imaging visit. Table S6 Odds ratio estimates for the association between CMR measurementsand carriership of a HCM variant in people without established cardiac disease at the time of imaging visit. Table S7 Odds ratio estimates for the association between CMR measurementsand carriership of a DCM variant in people without established cardiac disease at the time of imaging visit. Table S8 Baseline characteristics before and after imputation. Table S9 Exploring potential non-linear associations between CMR measurements and carriership of cardiomyopathy-associated variants using a multivariable model. Table S10 Sex-specific subgroup analyses for the association between CMR measurements and carriership of cardiomyopathy variants. Table S11 Age-specific subgroup analyses for the association between CMR measurements and carriership of cardiomyopathy variants. Table S12 Heterogeneity of the observed associations between CMR measurements and the three most common HCM and DCM genes. Table S13 Odds ratio estimates for the association between CMR measurements and the three most common HCM and DCM-associated genes. Table S14 Common genetic variants in or around known cardiomyopathy genes associated with CMR measurements in GWAS.

Additional file 2: Methods, Figure legends, Figures S1–S10. Methods—Additional methods. Figure legends—Full legends of Figs. S1–S10. Fig. S1 Loadings of the eight principal components explaining 90% of the variance in the CMR measurements. Fig. S2 Cumulative variance explained by the eight principal components explaining 90% of the CMR measurements. Fig. S3 Distribution of UK Biobank participants carrying pathogenic and likely pathogenic variants in cardiomyopathy-associated genes. Fig. S4 Survival of incident atrial fibrillation based on CMR measurements. Fig. S5 Survival of incident heart failure based on CMR measurements. Fig. S6 Spearman correlation between the effect estimates of the analyses on complete-case and imputed data. Fig. S7 Association of CMR measurements with HCM G + and the three most common HCM genes. Fig. S8 Association of CMR measurements with DCM G + and the three most common DCM genes. Fig. S9 Association of CMR measurements with the most common HCM genes. Fig. S10 Association of CMR measurements with the most common DCM genes.

## Data Availability

Analyses were conducted using python 3.11. For full code availability see repository: https://gitlab.com/mvvugt/cmr_geno_pub. The data used for this can be applied for with the UK biobank.

## Abbreviations

AF: Atrial fibrillation
CMP: Cardiomyopathy
CMR: Cardiac magnetic resonance imaging
DL: Deep learning
DCM: Dilated cardiomyopathy
EDV: End-diastolic volume
EF: Ejection fraction
ESV: End-systolic volume
GWAS: Genome-wide association study
G +: Participants carrying disease-associated variants
HCM: Hypertrophic cardiomyopathy
HF: Heart failure
HR: Hazard ratio
I: Indexed
IQR: Interquartile range
LA: Left atrial
LV: Left ventricular
MAPSE: Mitral annular plane systolic excursion
OR: Odds ratio
Pump: Pump volume
RA: Right atrial
Res: Reservoir volume
RV: Right ventricular
SV: Stroke volume
TAPSE: Tricuspid annular plane systolic excursion
V max: Maximal volume
V min: Minimal volume
WES: Whole exome sequencing
2Ch: 2-Chamber
4Ch: 4-Chamber
95%CI: 95% Confidence interval
