# Supplementary material for: Cardiac magnetic resonance markers of pre-clinical hypertrophic and dilated cardiomyopathy in genetic variant carriers
Source: BMC Med. 2025 Jul 15;23:421. doi: 10.1186/s12916-025-04226-4 (PMC12261545; doi:10.1186/s12916-025-04226-4)
Supplement: Supplementary file 2 — Additional file 2: Methods, Figure legends, Figures S1–S10. Methods—Additional methods. Figure legends—Full legends of Figs. S1–S10. Fig. S1 Loadings of the eight principal components explaining 90% of the variance in the CMR measurements. Fig. S2 Cumulative variance explained by the eight principal components explaining 90% of the CMR measurements. Fig. S3 Distribution of UK Biobank participants carrying pathogenic and likely pathogenic variants in cardiomyopathy-associated genes. Fig. S4 Survival of incident atrial fibrillation based on CMR measurements. Fig. S5 Survival of incident heart failure based on CMR measurements. Fig. S6 Spearman correlation between the effect estimates of the analyses on complete-case and imputed data. Fig. S7 Association of CMR measurements with HCM G + and the three most common HCM genes. Fig. S8 Association of CMR measurements with DCM G + and the three most common DCM genes. Fig. S9 Association of CMR measurements with the most common HCM genes. Fig. S10 Association of CMR measurements with the most common DCM genes. [file 12916_2025_4226_MOESM2_ESM.pdf]

Supplementary Materials

Content

Methods……………………………………………………………………………………..2
Figure legends…………………………………………...……………………………….3
Figure S1..…………………………………………………………………………………..6
Figure S2..…………………………………………………………………………………..7
Figure S3..…………………………………………………………………………………..8
Figure S4..…………………………………………………………………………………..9
Figure S5..…………………………………………………………………………………10
Figure S6..…………………………………………………………………………………11
Figure S7..…………………………………………………………………………………12
Figure S8..…………………………………………………………………………………13
Figure S9..…………………………………………………………………………………14
Figure S10…………………………………………………………………………………15

Methods

Data engineering strategy

We developed a de novo data engineering pipeline for extracting outcome data from the UK Biobank called “PhenotypeConstructor”. Its core functionality revolves around processing a range of disease definitions to extract the earliest diagnosis dates for various conditions. This includes interpreting ICD diagnosis codes, self-reported diagnoses, doctor-confirmed conditions, as well as medication details, and family health history. The phenotypes, corresponding UK Biobank field names, and codes considered in this study are described in **Supplementary Table S2**.

Figure legends

Figure S1. Loadings of the eight principal components explaining 90% of the variance in the CMR measurements.

Abbreviations: EF = ejection fraction, EDV = end-diastolic volume, ESV = end-systolic volume, i = indexed, LA = left atrial, LV = left ventricular, MAPSE = mitral annular plane systolic excursion, PC = principal component, RA = right atrial, RV = right ventricular, SV = stroke volume, TAPSE = tricuspid annular plane systolic excursion, V = volume, 2Ch = in 2-chamber view.

Figure S2. Cumulative variance explained by the eight principal components explaining 90% of the CMR measurements.

Figure S3. Distribution of UK biobank participants carrying pathogenic and likely pathogenic variants in cardiomyopathy-associated genes.

Figure S4. Survival of incident atrial fibrillation based on CMR measurements.

The curves estimate cumulative incidence for atrial fibrillation categorising the CMR measurements into two groups: an 85% 'reference' group and a 15% 'risk increasing' group, based on either the 15th or 85th percentile of CMR measurements as the cut-off point. Each plot is annotated with the hazard ratio derived from univariable Cox regression, along with the corresponding confidence interval and p-value.

Abbreviations: EF = ejection fraction, EDV = end-diastolic volume, ESV = end-systolic volume, i = indexed, LA = left atrial, LV = left ventricular, RA = right atrial, RV = right ventricular, SV = stroke volume, TAPSE = tricuspid annular plane systolic excursion, V = volume, 4Ch = in 4-chamber view.

Figure S5. Survival of incident heart failure based on CMR measurements.

The curves estimate cumulative incidence for heart failure categorising the CMR measurements into two groups: an 85% 'reference' group and a 15% 'risk increasing' group, based on either the 15th or 85th percentile of CMR measurements as the cut-off point. Each plot is annotated with the hazard ratio derived from univariable Cox regression, along with the corresponding confidence interval and p-value.

Abbreviations: EF = ejection fraction, EDV = end-diastolic volume, ESV = end-systolic volume, i = indexed, LA = left atrial, LV = left ventricular, RA = right atrial, RV = right ventricular, SV = stroke volume, TAPSE = tricuspid annular plane systolic excursion, V = volume, 4Ch = in 4-chamber view.

Figure S6. Spearman correlation between the effect estimates of the analyses on complete-case and imputed data.

Figure S7. Association of CMR measurements with HCM G+ and the three most common HCM genes.

Associations are presented as $-\log_{10}$(p-value) multiplied by the effect direction. Results with a p-value smaller than 6.25×10^{-3} are indicated by a star and smaller than 0.05 are indicated with a diamond. Heterogeneity p-values did not reach statistical significance for any of the CMR measurements.

Abbreviations: CMR = cardiac magnetic resonance imaging, EF = ejection fraction, ESV = end-systolic volume, HCM = hypertrophic cardiomyopathy, i = indexed, RA = right atrial, RV = right ventricular, TAPSE = tricuspid annular plane systolic excursion, V = volume, 4Ch = in 4-chamber view.

Figure S8. Association of CMR measurements with DCM G+ and the three most common DCM genes.

Associations are presented as $-\log_{10}$(p-value) multiplied by the effect direction. Results with a p-value smaller than 6.25×10^{-3} are indicated by a star and smaller than 0.05 are indicated with a diamond. Heterogeneity p-values reached statistical significance for all of the CMR measurements.

Abbreviations: CMR = cardiac magnetic resonance imaging, DCM = dilated cardiomyopathy, EF = ejection fraction, ESV = end-systolic volume, i = indexed, LV = left ventricular.

Figure S9. Association of CMR measurements with the most common HCM genes.

None of the associations of CMR measurements with *MYH7* reached statistical significance.

Abbreviations: CMR = cardiac magnetic resonance imaging, EF = ejection fraction, EDV = end-diastolic volume, ESV = end-systolic volume, HCM = hypertrophic cardiomyopathy, i = indexed, LV = left ventricular, MAPSE = mitral annular plane systolic excursion, OR = odds ratio, RV = right ventricular, TAPSE = tricuspid annular plane systolic excursion, 4Ch = in 4-chamber view, 95%CI = 95% confidence interval.

Figure S10. Association of CMR measurements with the most common DCM genes.

None of the associations of CMR measurements with *FLNC* reached statistical significance.

Abbreviations: CMR = cardiac magnetic resonance imaging, DCM = dilated cardiomyopathy, EF = ejection fraction, ESV = end-systolic volume, i = indexed, LV = left ventricular, MAPSE = mitral annular plane systolic excursion, OR = odds ratio, pump = pump volume, 2Ch = in 2-chamber view, 95%CI = 95% confidence interval.

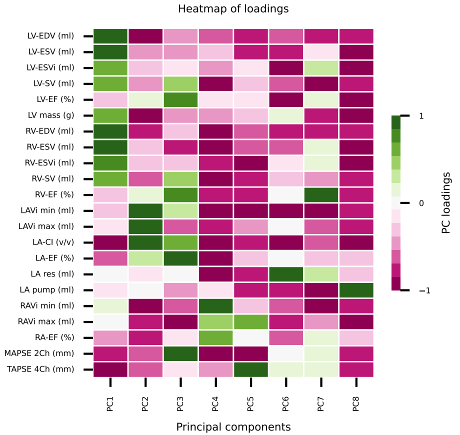

Heatmap of loadings
LV-EDV (ml)
LV-ESV (ml)
LV-ESVi (ml)
LV-SV (ml)
LV-EF (%)
LV mass (g)
RV-EDV (ml)
RV-ESV (ml)
RV-ESVi (ml)
RV-SV (ml)
RV-EF (%)
LAVi min (ml)
LAVi max (ml)
LA-CI (v/v)
LA-EF (%)
LA res (ml)
LA pump (ml)
RAVi min (ml)
RAVi max (ml)
RA-EF (%)
MAPSE 2Ch (mm)
TAPSE 4Ch (mm)
PC1
PC2
PC3
PC4
PC5
PC6
PC7
PC8
Principal components
1
0
−1
PC loadings

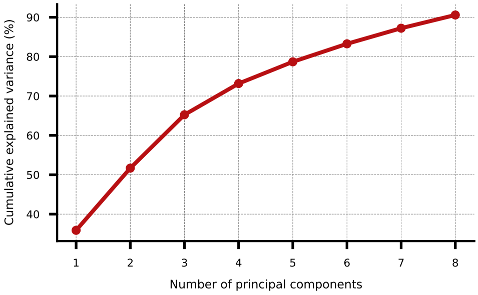

Cumulative explained variance (%)
90
80
70
60
50
40
1
2
3
4
5
6
7
8
Number of principal components

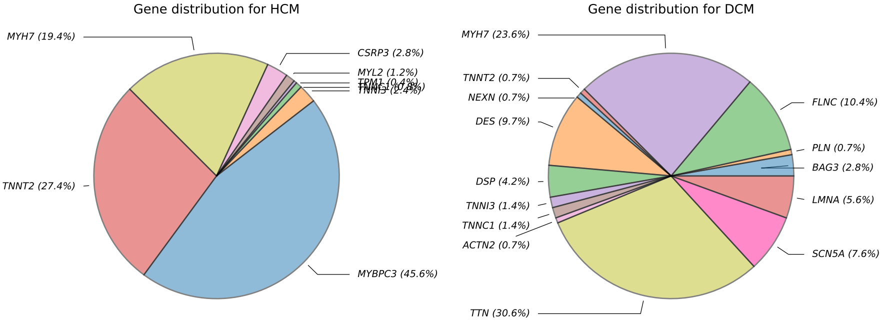
Gene distribution for HCM
MYH7 (19.4%)
CSRP3 (2.8%)
MYL2 (1.2%)
TPM1 (0.4%)
TNNI3 (2.4%)
TNNT2 (27.4%)
MYBPC3 (45.6%)
Gene distribution for DCM
MYH7 (23.6%)
TNNT2 (0.7%)
NEXN (0.7%)
DES (9.7%)
DSP (4.2%)
TNNI3 (1.4%)
TNNC1 (1.4%)
ACTN2 (0.7%)
TTN (30.6%)
FLNC (10.4%)
PLN (0.7%)
BAG3 (2.8%)
LMNA (5.6%)
SCN5A (7.6%)

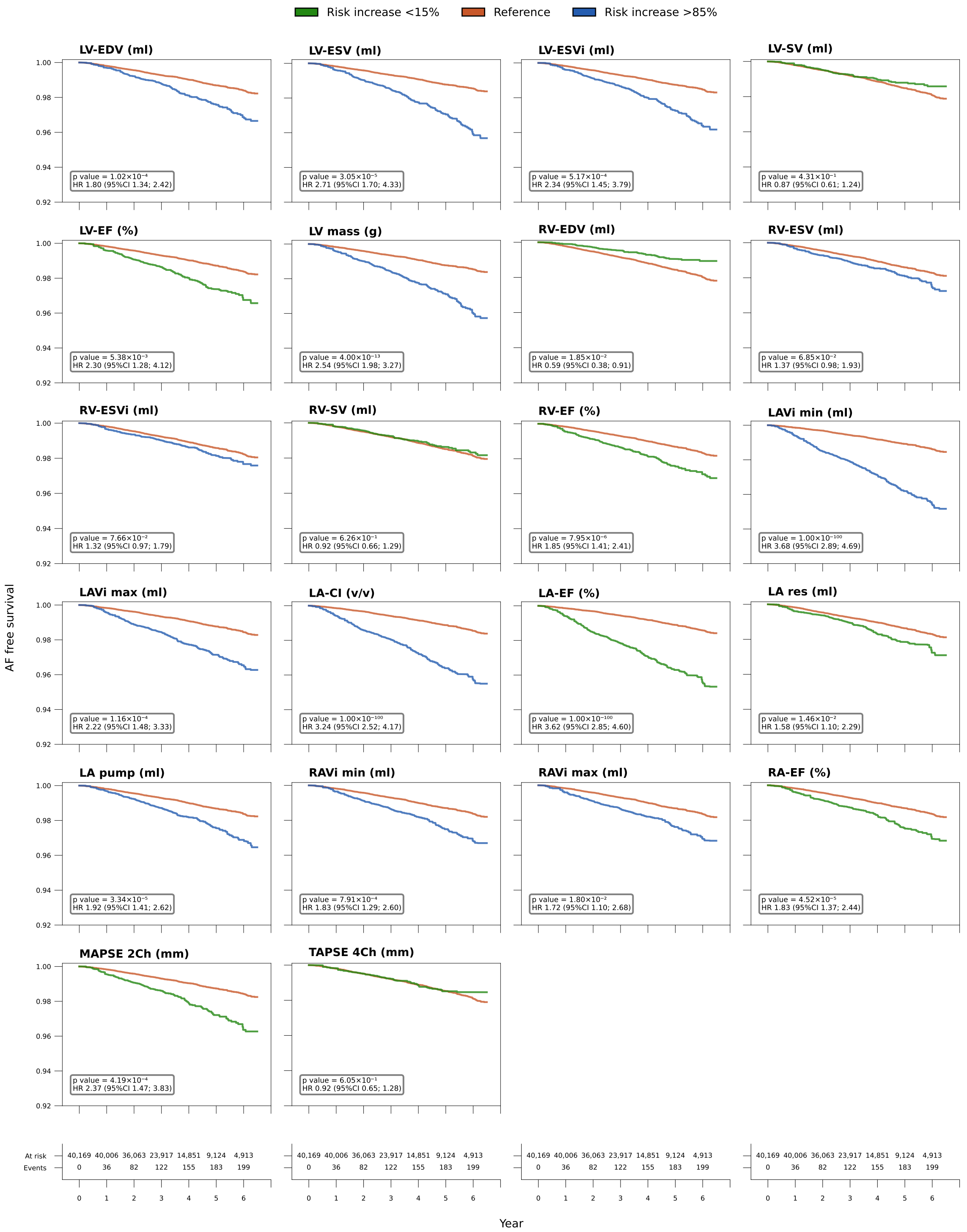
Risk increase <15%
Reference
Risk increase >85%
AF free survival
LV-EDV (ml)
p value = 1.02×10^{-4}
HR 1.80 (95%CI 1.34; 2.42)
LV-ESV (ml)
p value = 3.05×10^{-5}
HR 2.71 (95%CI 1.70; 4.33)
LV-ESVi (ml)
p value = 5.17×10^{-4}
HR 2.34 (95%CI 1.45; 3.79)
LV-SV (ml)
p value = 4.31×10^{-1}
HR 0.87 (95%CI 0.61; 1.24)
LV-EF (%)
p value = 5.38×10^{-3}
HR 2.30 (95%CI 1.28; 4.12)
LV mass (g)
p value = 4.00×10^{-13}
HR 2.54 (95%CI 1.98; 3.27)
RV-EDV (ml)
p value = 1.85×10^{-2}
HR 0.59 (95%CI 0.38; 0.91)
RV-ESV (ml)
p value = 6.85×10^{-2}
HR 1.37 (95%CI 0.98; 1.93)
RV-ESVi (ml)
p value = 7.66×10^{-2}
HR 1.32 (95%CI 0.97; 1.79)
RV-SV (ml)
p value = 6.26×10^{-1}
HR 0.92 (95%CI 0.66; 1.29)
RV-EF (%)
p value = 7.95×10^{-6}
HR 1.85 (95%CI 1.41; 2.41)
LAVi min (ml)
p value = 1.00×10^{-100}
HR 3.68 (95%CI 2.89; 4.69)
LAVi max (ml)
p value = 1.16×10^{-4}
HR 2.22 (95%CI 1.48; 3.33)
LA-CI (v/v)
p value = 1.00×10^{-100}
HR 3.24 (95%CI 2.52; 4.17)
LA-EF (%)
p value = 1.00×10^{-100}
HR 3.62 (95%CI 2.85; 4.60)
LA res (ml)
p value = 1.46×10^{-2}
HR 1.58 (95%CI 1.10; 2.29)
LA pump (ml)
p value = 3.34×10^{-5}
HR 1.92 (95%CI 1.41; 2.62)
RAVi min (ml)
p value = 7.91×10^{-4}
HR 1.83 (95%CI 1.29; 2.60)
RAVi max (ml)
p value = 1.80×10^{-2}
HR 1.72 (95%CI 1.10; 2.68)
RA-EF (%)
p value = 4.52×10^{-5}
HR 1.83 (95%CI 1.37; 2.44)
MAPSE 2Ch (mm)
p value = 4.19×10^{-4}
HR 2.37 (95%CI 1.47; 3.83)
TAPSE 4Ch (mm)
p value = 6.05×10^{-1}
HR 0.92 (95%CI 0.65; 1.28)
1.00
0.98
0.96
0.94
0.92
At risk
Events
40,169 40,006 36,063 23,917 14,851 9,124 4,913
0 36 82 122 155 183 199
0 1 2 3 4 5 6
Year

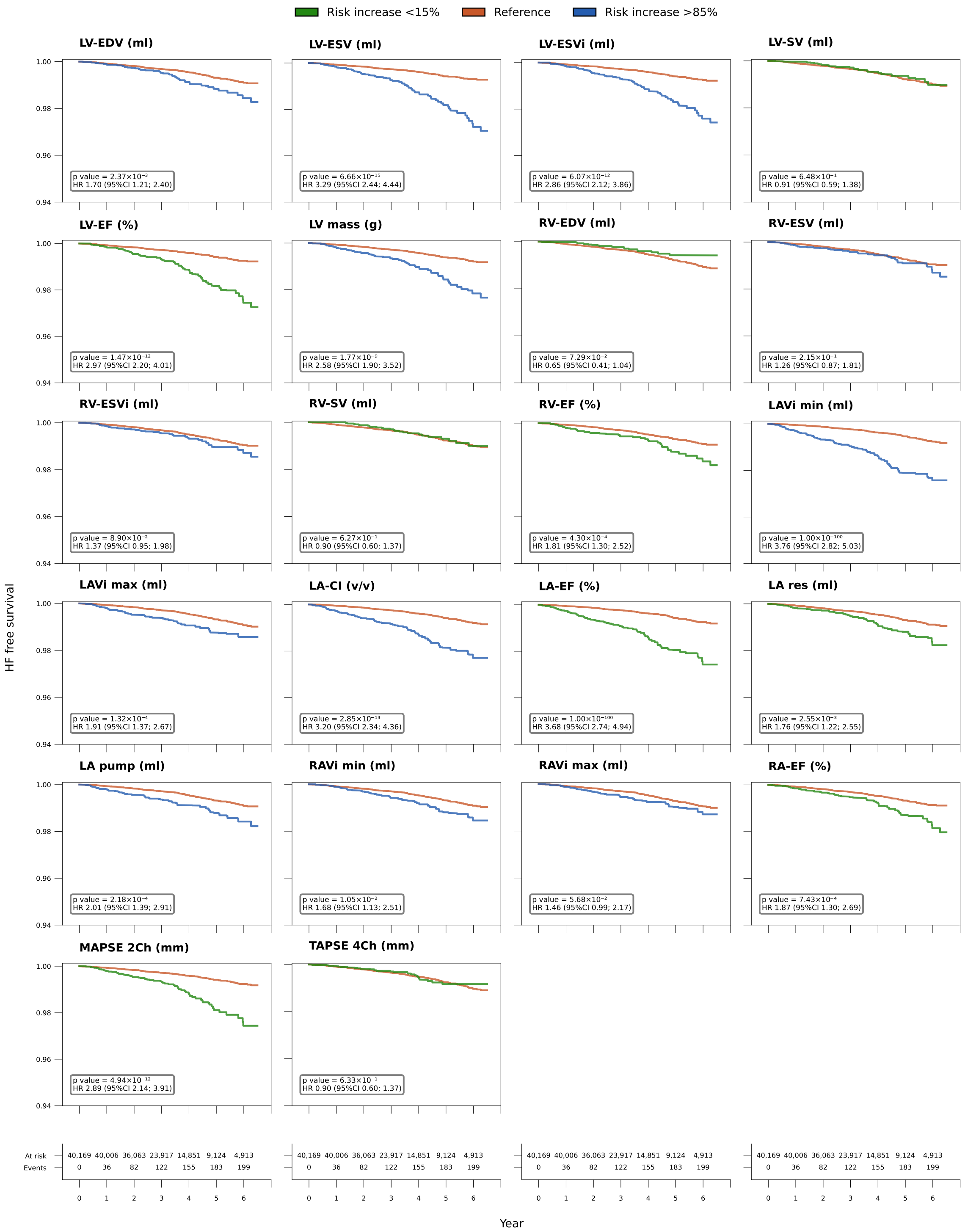
Risk increase <15%
Reference
Risk increase >85%
HF free survival
LV-EDV (ml)
p value = 2.37×10^{-3}
HR 1.70 (95%CI 1.21; 2.40)
LV-ESV (ml)
p value = 6.66×10^{-15}
HR 3.29 (95%CI 2.44; 4.44)
LV-ESVi (ml)
p value = 6.07×10^{-12}
HR 2.86 (95%CI 2.12; 3.86)
LV-SV (ml)
p value = 6.48×10^{-1}
HR 0.91 (95%CI 0.59; 1.38)
LV-EF (%)
p value = 1.47×10^{-12}
HR 2.97 (95%CI 2.20; 4.01)
LV mass (g)
p value = 1.77×10^{-9}
HR 2.58 (95%CI 1.90; 3.52)
RV-EDV (ml)
p value = 7.29×10^{-2}
HR 0.65 (95%CI 0.41; 1.04)
RV-ESV (ml)
p value = 2.15×10^{-1}
HR 1.26 (95%CI 0.87; 1.81)
RV-ESVi (ml)
p value = 8.90×10^{-2}
HR 1.37 (95%CI 0.95; 1.98)
RV-SV (ml)
p value = 6.27×10^{-1}
HR 0.90 (95%CI 0.60; 1.37)
RV-EF (%)
p value = 4.30×10^{-4}
HR 1.81 (95%CI 1.30; 2.52)
LAVi min (ml)
p value = 1.00×10^{-100}
HR 3.76 (95%CI 2.82; 5.03)
LAVi max (ml)
p value = 1.32×10^{-4}
HR 1.91 (95%CI 1.37; 2.67)
LA-CI (v/v)
p value = 2.85×10^{-13}
HR 3.20 (95%CI 2.34; 4.36)
LA-EF (%)
p value = 1.00×10^{-100}
HR 3.68 (95%CI 2.74; 4.94)
LA res (ml)
p value = 2.55×10^{-3}
HR 1.76 (95%CI 1.22; 2.55)
LA pump (ml)
p value = 2.18×10^{-4}
HR 2.01 (95%CI 1.39; 2.91)
RAVi min (ml)
p value = 1.05×10^{-2}
HR 1.68 (95%CI 1.13; 2.51)
RAVi max (ml)
p value = 5.68×10^{-2}
HR 1.46 (95%CI 0.99; 2.17)
RA-EF (%)
p value = 7.43×10^{-4}
HR 1.87 (95%CI 1.30; 2.69)
MAPSE 2Ch (mm)
p value = 4.94×10^{-12}
HR 2.89 (95%CI 2.14; 3.91)
TAPSE 4Ch (mm)
p value = 6.33×10^{-1}
HR 0.90 (95%CI 0.60; 1.37)
1.00
0.98
0.96
0.94
At risk 40,169 40,006 36,063 23,917 14,851 9,124 4,913
Events 0 36 82 122 155 183 199
0 1 2 3 4 5 6
Year

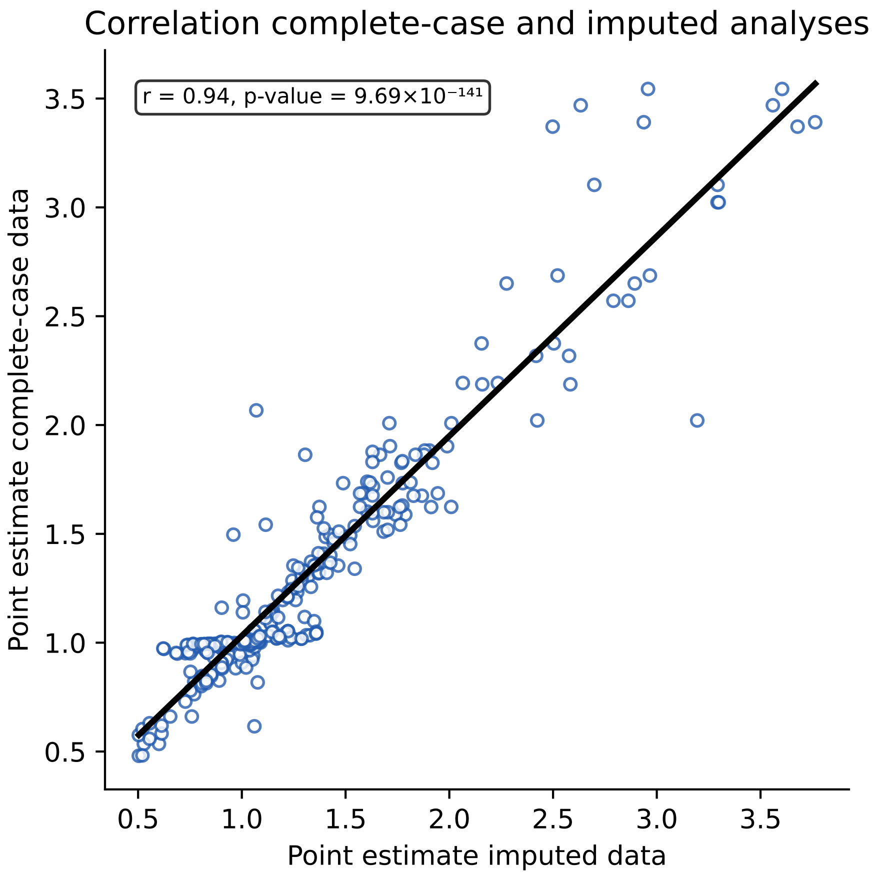
Correlation complete-case and imputed analyses
r = 0.94, p-value = 9.69×10⁻¹⁴¹
Point estimate complete-case data
Point estimate imputed data
0.5
1.0
1.5
2.0
2.5
3.0
3.5

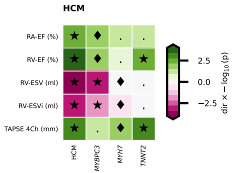
HCM
RA-EF (%)
RV-EF (%)
RV-ESV (ml)
RV-ESVi (ml)
TAPSE 4Ch (mm)
HCM
MYBPC3
MYH7
TNNT2
2.5
0.0
−2.5
dir × −log10 (p)

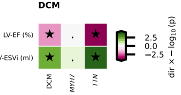

DCM
LV-EF (%)
LV-ESVi (ml)
DCM
MYH7
TTN
2.5
0.0
−2.5
dir × −log10 (p)

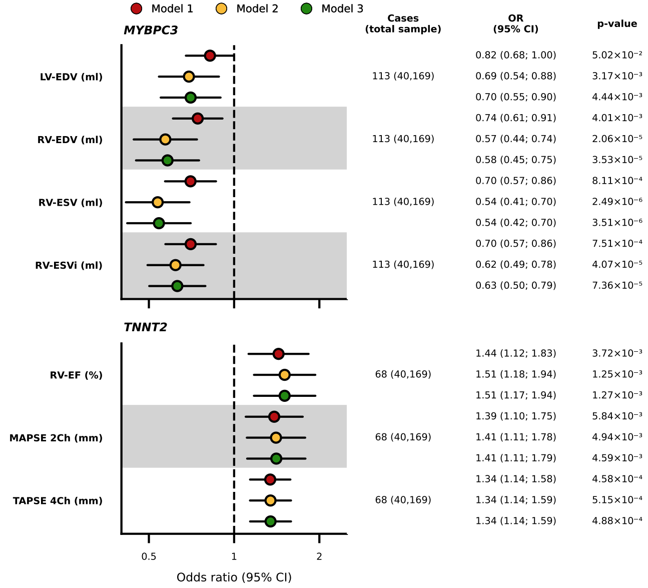
Model 1
Model 2
Model 3
MYBPC3
Cases (total sample)
OR (95% CI)
p-value
LV-EDV (ml)
113 (40,169)
0.82 (0.68; 1.00) 5.02×10^{-2}
0.69 (0.54; 0.88) 3.17×10^{-3}
0.70 (0.55; 0.90) 4.44×10^{-3}
RV-EDV (ml)
113 (40,169)
0.74 (0.61; 0.91) 4.01×10^{-3}
0.57 (0.44; 0.74) 2.06×10^{-5}
0.58 (0.45; 0.75) 3.53×10^{-5}
RV-ESV (ml)
113 (40,169)
0.70 (0.57; 0.86) 8.11×10^{-4}
0.54 (0.41; 0.70) 2.49×10^{-6}
0.54 (0.42; 0.70) 3.51×10^{-6}
RV-ESVi (ml)
113 (40,169)
0.70 (0.57; 0.86) 7.51×10^{-4}
0.62 (0.49; 0.78) 4.07×10^{-5}
0.63 (0.50; 0.79) 7.36×10^{-5}
TNNT2
RV-EF (%)
68 (40,169)
1.44 (1.12; 1.83) 3.72×10^{-3}
1.51 (1.18; 1.94) 1.25×10^{-3}
1.51 (1.17; 1.94) 1.27×10^{-3}
MAPSE 2Ch (mm)
68 (40,169)
1.39 (1.10; 1.75) 5.84×10^{-3}
1.41 (1.11; 1.78) 4.94×10^{-3}
1.41 (1.11; 1.79) 4.59×10^{-3}
TAPSE 4Ch (mm)
68 (40,169)
1.34 (1.14; 1.58) 4.58×10^{-4}
1.34 (1.14; 1.59) 5.15×10^{-4}
1.34 (1.14; 1.59) 4.88×10^{-4}
0.5
1
2
Odds ratio (95% CI)

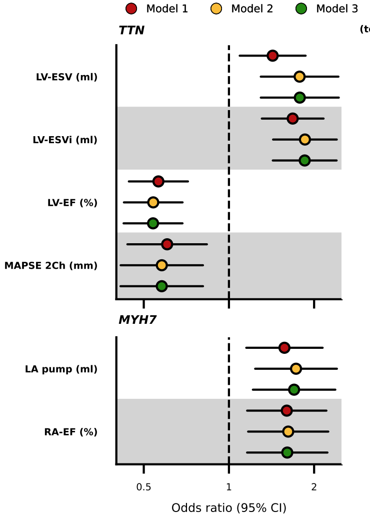

Gene	Trait	Model	Cases (total sample)	OR (95% CI)	p-value
TTN	LV-ESV (ml)	Model 1	44 (40,169)	1.43 (1.09; 1.87)	9.35×10^{-3}
		Model 2		1.78 (1.30; 2.44)	3.62×10^{-4}
		Model 3		1.78 (1.30; 2.45)	3.72×10^{-4}
	LV-ESVi (ml)	Model 1	44 (40,169)	1.68 (1.31; 2.16)	5.23×10^{-5}
		Model 2		1.86 (1.43; 2.41)	2.94×10^{-6}
		Model 3		1.85 (1.43; 2.40)	3.30×10^{-6}
	LV-EF (%)	Model 1	44 (40,169)	0.56 (0.44; 0.72)	2.96×10^{-6}
		Model 2		0.54 (0.43; 0.69)	4.28×10^{-7}
		Model 3		0.54 (0.42; 0.69)	4.54×10^{-7}
	MAPSE 2Ch (mm)	Model 1	44 (40,169)	0.60 (0.44; 0.84)	2.32×10^{-3}
		Model 2		0.58 (0.41; 0.81)	1.39×10^{-3}
		Model 3		0.58 (0.41; 0.81)	1.42×10^{-3}
MYH7	LA pump (ml)	Model 1	34 (40,169)	1.57 (1.15; 2.14)	4.39×10^{-3}
		Model 2		1.73 (1.24; 2.41)	1.30×10^{-3}
		Model 3		1.70 (1.21; 2.38)	1.96×10^{-3}
	RA-EF (%)	Model 1	34 (40,169)	1.60 (1.16; 2.21)	4.27×10^{-3}
		Model 2		1.62 (1.17; 2.24)	3.77×10^{-3}
		Model 3		1.61 (1.16; 2.23)	4.45×10^{-3}
